# Diagnostic point-of-care ultrasound in obstetric anesthesia and critical care: a scoping review protocol

**DOI:** 10.1186/s13643-024-02673-3

**Published:** 2024-10-24

**Authors:** Ana Sjaus, Laura V. Young

**Affiliations:** 1 Department of Anesthesia, Pain Management and Perioperative Medicine, Dalhousie University, Halifax, NS Canada; 2 Department of Women’s and Obstetric Anesthesia, IWK Health, Halifax, NS Canada

**Keywords:** Point-of-care ultrasound, POCUS, Obstetric anesthesia, Obstetric critical care, Obstetrics, Pregnancy, Gastric ultrasound, Lung ultrasound, Echocardiography

## Abstract

**Background:**

Point-of-care ultrasound (POCUS) has gained popularity as a bedside diagnostic imaging modality. In obstetrical populations, particularly in acute care settings, POCUS serves as a valuable complement to clinical assessment. Despite its many applications, only a few have been defined and validated in the obstetric population. This scoping review aims to delineate literature on the diagnostic applications of POCUS in obstetric anesthesia and critical care.

**Methods:**

This review will adhere to the Joanna Briggs Institute methodology for scoping reviews, as updated by Arksey and O’Malley and in stages elaborated by Levac et al. Relevant literature will be identified using Medical Subject Headings (MeSH), keyword, and proximity searches and combined using Boolean operators in PubMed, Embase, and Web of Science from January 1, 2000, to the present. Two independent reviewers will screen literature against predefined eligibility criteria in abstract and full-text forms. A third reviewer will be consulted if consensus cannot be reached. Data extraction will be systematic, focusing on pre-specified variables aligned with the review’s aims. Descriptive statistical and thematic analysis will follow data extraction, with findings presented in graphical and tabular forms. The reporting will follow Preferred Reporting Items for Systematic reviews and Meta-Analysis extension for Scoping Reviews (PRISMA-ScR).

**Conclusion:**

This review will present the scope of the current literature on diagnostic POCUS in obstetric anesthesia and critical care, highlighting both strengths and gaps in existing knowledge. The insights gained will support future research, knowledge synthesis, and development of educational programs. The findings will be disseminated through peer-reviewed journal publications, conferences, and social media platforms.

**Systematic review registration:**

Not applicable.

**Supplementary Information:**

The online version contains supplementary material available at 10.1186/s13643-024-02673-3.

## Introduction

Point-of-care ultrasound (POCUS) allows acquisition of patient-specific and context-specific diagnostic information at the bedside and in real time. Initially adopted by emergency medicine, ultrasound-based point-of-care diagnostic techniques have rapidly diffused across acute care specialties such as critical care and anesthesiology, with recent uptake in other medical fields including general practice [1, 2].

Narrative and systematic reviews highlight the relevance and utility of point-of-care diagnostic ultrasound in the obstetrical population [3–5]. Pregnancy, labor, and delivery present unique clinical challenges for which POCUS, as a readily available diagnostic modality, is particularly well suited. Clinicians providing obstetric care are aware of the potential for young, healthy parturient transitioning rapidly to a severely compromised state. Events such as obstetric hemorrhage or embolic events can precipitate severe hemodynamic instability. Similarly, hypertensive disorders of pregnancy are associated with significant morbidity and end-organ dysfunction. Diagnostic POCUS, when used as an adjunct to clinical assessment, may enhance diagnostic speed and accuracy, thereby guiding treatment more effectively.

However, the anatomical and physiological changes of pregnancy may render standard interpretations from other populations inapplicable. For example, pregnancy-specific cutoff points and indices may be necessary for both normal baseline features as well as pathologies. The evidence regarding the role, baseline values, technical aspects, and patient benefits of many POCUS applications in obstetrical populations remain insufficiently defined.

Pending pregnancy-specific validation and standardization of techniques adopted from other contexts, the value of POCUS, and the increasing portability and accessibility of ultrasound technology have resulted in its growing popularity. Its applications have expanded beyond cardiac, gastric, and lung ultrasound, to include techniques such as transcranial Doppler and optic nerve sheath ultrasound [6, 7]. POCUS has been integrated into training programs, with a growing number of anesthesiologists including the obstetric subspecialty subset, becoming skilled in its use.

The objective of this review is to comprehensively present the scope and the nature of the existing literature on diagnostic POCUS in obstetric anesthesia and critical care. The intent is to identify research gaps and facilitate the work of researchers, clinicians, and educators to expedite further projects, define the scope of POCUS skills for obstetric anesthesiologists, develop curricula, and perform periodic updates.

## Methods

POCUS in obstetric anesthesiology and critical care is an emerging concept encompassing numerous applications. The literature is expected to be heterogeneous in focus and methodology. Therefore, following the indications and methodological considerations described initially by Arksey and O’Malley, and later Levac, a scoping review methodology is appropriate to map and broadly summarize the literature to date [8, 9].

The proposed scoping review will be conducted in accordance with the Joanna Briggs Institute (JBI) methodology for scoping reviews, in stages defined by Peters et al. [10]. The Preferred Reporting Items for Systematic reviews and Meta-Analysis extension for Scoping Reviews (PRISMA-ScR) will be used for presenting the results.^11^

### Stage 1: Identifying the research question

This review aims to explore the current scope of literature pertaining to diagnostic use of POCUS in obstetric anesthesia. Specifically, it will examine how POCUS has been used, studied, and the geographic origins of the work.

Objectives are as follows:

- To identify publications on diagnostic uses of POCUS in obstetric populations that are relevant to obstetric anesthesia and critical care, specifically those describing or embodying POC applications as defined below
- To identify the clinical aspects of POCUS applications including indications, assessments, and the utility of POCUS in selected publications
- To identify the technical aspects such as technologies and modes of ultrasound in the selected publications
- To summarize study methodologies, identify the geographical centers of knowledge generation, and estimate the potential impact of publications
- To identify gaps in the literature and make suggestions for future research

### Stage 2: Identifying relevant studies

#### Eligibility criteria

A summary of the inclusion and exclusion criteria is presented in Table 1. This scoping review will consider studies conducted from January 1, 2000, to the present, reflecting the relatively recent advent of POCUS. The review will include experimental study designs, randomized controlled trials, feasibility/validation studies, narrative and systematic reviews, descriptive observational designs, and case reports. Publications focused on POCUS education and simulation will be excluded.

**Table 1:** Eligibility criteria for inclusion in the review

	**Inclusion criteria**	**Exclusion criteria**
Publication date	January 1, 2000-present	Prior to January 1, 2000
Language	English	Non-English
Application and protocol	Diagnostic POCUS applications not limited to ocular (ONSD), transcranial Doppler, lung, cardiac, IVC, FAST (for intra-abdominal fluid), gastric, abdominal, airway, volume assessment, SVC, TTE, TEE, Doppler vein DVT for venous ultrasound, renal vascular, cardiac output, portal vein, VExUS	Interventional POCUS (neuraxial, regional anesthesia, & vascular access)
Source	Randomized controlled trials, feasibility studies, reviews, observational studies, validation studies, individual case reports	POCUS education/simulation studies
Population	Pregnant, parturient, and postpartum	Obstetric ultrasound for fetal indications, gynecologic ultrasound
Period	Third trimester, peripartum	

*ONSD* Optic nerve sheath diameter, *IVC* Inferior vena cava, *FAST* Focused assessment with sonography in trauma, *SVC *Superior vena cava, *TTE *Transthoracic echocardiogram, *TEE *Transesophageal echocardiogram, *DVT* Deep vein thrombosis, *VExUS* Venous excess ultrasound score

The gestational period of interest spans from the 24th week of pregnancy to the end of the peripartum period (6-week post-partum).

#### Conceptual framework and definition of POCUS

POCUS will be defined as “ultrasound intended for use at the bedside for immediate diagnostic purposes.” Given the novelty of the term POCUS, many publications may simply describe sonography. To distinguish POCUS from non-POC sonography, the I-AIM framework will be utilized. An application will be considered to embody POC if it meets the following criteria: it is indicated by a clinical question arising during anesthesia care or critical illness (I — indication) and requires bedside acquisition (A — acquire) and interpretation (I — interpret) skills that could be reasonably expected from a non-expert sonographer, such as the attending physician who will use the diagnostic information to make a medical decision [12]. This distinction may be challenging, particularly in cardiac sonography, as non-cardiologists are increasingly trained in advanced ultrasound techniques and the use of full-featured ultrasound equipment.

The POCUS applications of interest for this review include, but are not limited to, the following: ocular (optic nerve sheath diameter [ONSD]), transcranial Doppler, lung, cardiac, inferior vena cava (IVC), abdominal, gastric, airway, superior vena cava (SVC), focused assessment with sonography in trauma (FAST), transthoracic (TTE), transesophageal echocardiography (TEE), vascular venous ultrasound (for diagnosis of deep vein thrombosis (DVT)), portal vein, and venous excess ultrasound score (VExUS) and arterial (aortic, carotid, renal) applications.

Studies featuring obstetric ultrasound for fetal indications (uterine, placental, or fetal) and gynecological ultrasound as well as procedural POCUS (for neuraxial or regional anesthesia and vascular access) will be excluded.

#### Definitions

This review will use the following definitions:

- “POCUS application”: A well-described use of POCUS focused on a specific organ system or anatomical structure (e.g., cardiac, gastric, lung)
- “Ultrasound mode”: A specific utility of ultrasound machines that provides the technical basis for certain types of ultrasound imaging, such as M-mode, Doppler ultrasound, and 2D ultrasound
- “Indication”: A differential/working diagnosis, sign, symptom, or finding that prompts the use of imaging
- “Assessment”: A specific ultrasound-based technique or procedure for evaluating a particular aspect of organ function (for example, cardiac output) or specific pathology
- “Utility”: The value or function of ultrasound in a study or clinical case, excluding point estimates of effect sizes.

#### Search strategy

A medical librarian experienced in literature retrieval for knowledge synthesis will assist in developing and implementing the bibliographic database searches.

Key words and index terms of a sample of the most relevant retrieved articles found in the titles and abstracts will be analyzed to develop a comprehensive search strategy. This search strategy will use all identified keywords and index terms to perform a search PubMed, Embase, and Web of Science (Supplementary file 1). All search terms will adhere to major headings specific to the database: MeSH for PubMed and Emtree for Embase. Since Web of Science does not use major headings, the search will employ individual search terms and major subheadings as search phrases. A broad search combining major headings and keyword terms for “obstetric,” “ultrasound,” “POCUS,” and “anesthesia/critical care” will be conducted (combined using AND) and then refined to address the heterogenous terminology by including “applications,” “assessments,” and “protocols” with Boolean operators. Manual ascendancy and descendancy searching of reference lists in the included articles will be performed to identify additional publications. The search strategy, protocol, and search concepts are detailed in Supplementary file 2 using PubMed as an example.

Validation of search results will be conducted using benchmarking to 10–15% relevance and by ensuring inclusion of a set of pre-identified “landmark” studies. Adjustments may be required to make the search more specific or to broaden the search, depending on the results of the validation procedure.

### Stage 3: Study selection

The final search results for screening will be exported into Covidence for duplicate removal (Covidence systematic review software, Veritas Health Innovation, Melbourne, Australia, available at www.covidence.org). Subsequently, titles and abstracts will be screened by two independent reviewers who are familiar with objectives, relevant definitions, and inclusion and exclusion criteria. Any discrepancies will be resolved through discussion. If consensus cannot be reached, a third reviewer will be consulted. To fine-tune inclusion/exclusion criteria, an assessment of the level of agreement will be conducted after the first 10% of the studies have been screened by both reviewers. If a high level of agreement (> 90%) is not achieved, the research team will discuss the reasons in detail and review the inclusion criteria with the possibility of revision and clarification.

Studies that pass the screening will be retrieved in full-text form. The full text of selected citations will be assessed in detail against the inclusion criteria. Any remaining duplicate studies published in different journals will be merged. The process will be reported in full and presented in a PRISMA-ScR flow diagram (Supplementary file 3).

### Stage 4: Charting the data

Data extraction will be conducted by a minimum of two independent reviewers utilizing an adapted standardized data extraction template, as per the JBI methodology guidance for scoping reviews [10]. Supplementary file 4 lists the extracted variables, their values/categories, and instructions for the extracting researchers. The Covidence data extraction tool will be customized for this project to ensure uniform data extraction by all researchers.

Prior to formal data extraction, the team will pilot the data extraction tool and analyze a sample of studies (3–4 from each methodological type selected for full-text review). Necessary adjustments, including the addition or removal of variables and refinement of categories, will be made based on this pilot phase.

Data extraction agreement and quality assessment will be performed at 10%, 50%, and 100% of the selected studies. To ensure transparency, all team communications, including consensus decisions, adjustments, and changes to the process, will be documented and reported separately.

### Stage 5: Collating, summarizing, and reporting of results

Data will be exported into a format compatible with Stata/IC 16.1, rev. 06/2023 (Stata Corp., College Station, TX, USA). Descriptive statistics will be used to present the findings, which will be reported following the PRISMA-ScR guidelines [11].

Results will be organized in tables and presented as temporal trends in overall publication, stratified by publication characteristics, methodology, clinical aspects, and technology. Multidimensional, mixed attribute plots (e.g., bubble plots, Nightingale rose plot, stacked histograms) will be used to illustrate the findings in a visually clear and accessible form.

## Discussion

This review will present the original effort to summarize the body of literature on diagnostic point-of-care ultrasound (POCUS) in maternal anesthesia and critical care for obstetric populations. Given the anticipated overlap with the literature on fetal and procedural ultrasound, a conceptual framework was employed to delineate diagnostic POCUS, even when not explicitly defined as such in the source publications. Consequently, this review aims to capture a broad spectrum of research and clinical reports.

Diagnostic POCUS is inherently user and context dependent. The clinical context provided in case reports, combined with original research, will offer a comprehensive exposition of the relevance and gaps in the current research landscape.

Quality assessment is not an integral component of scoping reviews. The heterogeneity of methodologies among included publications renders such an endeavor unfeasible, which presents a limitation of this review. Despite rigorous systematic searching, some relevant publications may be missed. Additionally, case reports are subject to publication bias, as cases where POCUS was not beneficial are less likely to be reported. Moreover, despite efforts to clearly define POCUS, we expect to encounter publications that may not specify all aspects outlined in our framework, potentially leading to misinterpretation. Despite these limitations, the review is poised to offer valuable insights into the current state of the literature.

## Conclusion

The scoping review will present an overview of the current literature on POCUS use in obstetric populations, identifying areas of strength and knowledge gaps. The findings, future directions, and implications for the scope of diagnostic POCUS in obstetric anesthesiology and maternal critical care will be discussed in the final report.

## Supplementary Information

Supplementary Material 1: Supplementary file 1. Search Protocol, Strategy, and Terms. A pilot comprehensive search in PubMed

Supplementary Material 2: Supplementary file 2. A pilot comprehensive search in PubMed﻿

Supplementary Material 3: Supplementary file 3. PRISMA Flow-chart

Supplementary Material 4: Supplementary file 4. POCUS in obstetric anesthesia data extraction instrument and codebook with guidance

## Data Availability

Not applicable.

## Abbreviations

DVT: Deep vein thrombosis
FAST: Focused assessment with sonography in trauma
IVC: Inferior vena cava
JBI: Joanna Briggs Institute
MeSH: Medical Subject Headings
ONSD: Optic nerve sheath diameter
POC: Point of care
POCUS: Point-of-care ultrasound
PRISMA-ScR: Preferred Reporting Items for Systematic reviews and Meta-Analysis extension for Scoping Reviews
SIG: Special interest group
SOAP: Society of Obstetric Anesthesia and Perinatology
SVC: Superior vena cava
TEE: Transesophageal echocardiogram
TTE: Transthoracic echocardiogram
VExUS: Venous excess ultrasound score

## References

[1] Frankel HL, Kirkpatrick AW, Elbarbary M, Blaivas M, Desai H, Evans D, et al. Guidelines for the appropriate use of bedside general and cardiac ultrasonography in the evaluation of critically ill patients-part I: general ultrasonography. Crit Care Med. 2015;43(11):2479–502. 26468699 10.1097/CCM.0000000000001216

[2] Arnold MJ, Jonas CE, Carter RE. Point-of-care ultrasonography. Am Fam Physician. 2020;101(5):275–85. 32109031

[3] Zieleskiewicz L, Bouvet L, Einav S, Duclos G, Leone M. Diagnostic point-of-care ultrasound: applications in obstetric anaesthetic management. Anaesthesia. 2018;73(10):1265–79. 30047997 10.1111/anae.14354

[4] Van de Putte P, Vernieuwe L, Bouchez S. Point-of-care ultrasound in pregnancy: gastric, airway, neuraxial, cardiorespiratory. Curr Opin Anaesthesiol. 2020;33(3):277–83. 32324656 10.1097/ACO.0000000000000846

[5] Simard C, Yang S, Koolian M, Shear R, Rudski L, Lipes J. The role of echocardiography in amniotic fluid embolism: a case series and review of the literature. Can J Anaesth J Can Anesth. 2021;68(10):1541–8. 10.1007/s12630-021-02065-4 34312822

[6] Mowafy SMS, Abd Ellatif SE. Transcranial Doppler role in prediction of post-dural puncture headache in parturients undergoing elective cesarean section: prospective observational study. J Anesth. 2019;33(3):426–34. 31073654 10.1007/s00540-019-02652-2

[7] Assu SM, Bhatia N, Jain K, Gainder S, Sikka P, Aditya AS. Sonographic optic nerve sheath diameter following seizure prophylaxis in pre-eclamptic parturients with severe features: a prospective, observational study. J Ultrasound Med Off J Am Inst Ultrasound Med. 2021;40(11):2451–7. 10.1002/jum.15632 33448448

[8] Arksey H, O’Malley L. Scoping studies: towards a methodological framework. Int J Soc Res Methodol Theory Pract. 2005;8(1):19–32.

[9] Levac D, Colquhoun H, O’Brien KK. Scoping studies: advancing the methodology. Implement Sci IS. 2010;20(5):69. 10.1186/1748-5908-5-69 PMC2954944 20854677

[10] Peters MDJ, Marnie C, Tricco AC, Pollock D, Munn Z, Alexander L, et al. Updated methodological guidance for the conduct of scoping reviews. JBI Evid Implement. 2021;19(1):3. 33570328 10.1097/XEB.0000000000000277

[11] Tricco AC, Lillie E, Zarin W, O’Brien KK, Colquhoun H, Levac D, et al. PRISMA extension for scoping reviews (PRISMA-ScR): checklist and explanation. Ann Intern Med. 2018;169(7):467–73. 30178033 10.7326/M18-0850

[12] Kruisselbrink R, Chan V, Cibinel GA, Abrahamson S, Goffi A. I-AIM (Indication, Acquisition, Interpretation, Medical decision-making) framework for point of care lung ultrasound. Anesthesiology. 2017;127(3):568–82. 28742530 10.1097/ALN.0000000000001779

