# Supplementary material for: Diagnostic point-of-care ultrasound in obstetric anesthesia and critical care: a scoping review protocol
Source: Syst Rev. 2024 Oct 24;13:268. doi: 10.1186/s13643-024-02673-3 (PMC11515486; doi:10.1186/s13643-024-02673-3)
Supplement: Supplementary file 1 — Supplementary Material 1: Supplementary file 1. Search Protocol, Strategy, and Terms. A pilot comprehensive search in PubMed [file 13643_2024_2673_MOESM1_ESM.docx]

Supplementary file 1: A pilot comprehensive search in PubMed

| **Date** | **Database** | **Search No.** | **Query** | **Search Result** |
| --- | --- | --- | --- | --- |
| 22/08/2023 | PubMed | #14 | #4 AND #9 Filters: Female, Adult: 19+ years, English, from 2000 - 2023 | [528](https://pubmed.ncbi.nlm.nih.gov/?term=%234+AND+%239&filter=sex.female&filter=age.alladult&filter=lang.english&filter=years.2000-2023&size=100&sort=relevance) |
|  |  | #13 | #4 AND #9 Filters: Female, Adult: 19+ years, from 2000 - 2023 | [594](https://pubmed.ncbi.nlm.nih.gov/?term=%234+AND+%239&filter=sex.female&filter=age.alladult&filter=years.2000-2023&size=100&sort=relevance) |
|  |  | #12 | #4 AND #9 Filters: Female, from 2000 - 2023 | [1,071](https://pubmed.ncbi.nlm.nih.gov/?term=%234+AND+%239&filter=sex.female&filter=years.2000-2023&size=100&sort=relevance) |
|  |  | #11 | #4 AND #9 Filters: from 2000 - 2023 | [1,399](https://pubmed.ncbi.nlm.nih.gov/?term=%234+AND+%239&filter=years.2000-2023&size=100&sort=relevance) |
|  |  | #10 | #4 AND #9 | [1,726](https://pubmed.ncbi.nlm.nih.gov/?term=%234+AND+%239&sort=relevance&size=100) |
|  |  | #9 | #5 OR #6 OR #7 OR #8 | [5,058,315](https://pubmed.ncbi.nlm.nih.gov/?term=%235+OR+%236+OR+%237+OR+%238&sort=relevance&size=100) |
|  |  | #8 | ((((((((((((((((((((((((((("stroke volume"[Title/Abstract]) OR ("cardiac output"[Title/Abstract])) OR ("ventric* function"[Title/Abstract:~4])) OR (contractil*[Title/Abstract])) OR (heart failure[Title/Abstract])) OR (cardiac failure[Title/Abstract])) OR (ventricular failure[Title/Abstract])) OR (pulmonary edema[Title/Abstract])) OR (lung edema[Title/Abstract])) OR (pulmonary congestion[Title/Abstract])) OR (lung water[Title/Abstract])) OR (pneumothorax[Title/Abstract])) OR (tamponade[Title/Abstract])) OR (effusion[Title/Abstract])) OR (emboli*[Title/Abstract])) OR (hemodynamic[Title/Abstract])) OR (hypotensi*[Title/Abstract])) OR (respiratory failure[Title/Abstract])) OR (hypoxi*[Title/Abstract])) OR (Oliguri*[Title/Abstract])) OR ("renal failure"[Title/Abstract:~6])) OR ("venous congestion"[Title/Abstract:~6])) OR (Intracranial hypertension[Title/Abstract])) OR (intracranial pressure[Title/Abstract])) OR (Cerebral edema[Title/Abstract])) OR (intubation[Title/Abstract])) OR (laryngosco*[Title/Abstract])) OR ((("Intubation, Intratracheal"[MeSH]) OR "Laryngoscopy"[MeSH]) OR "Airway Management"[MeSH]) OR ("Cardiac Output"[MeSH Terms] OR "Stroke Volume"[MeSH Terms] OR "Ventricular Function"[MeSH Terms] OR "ventricular function, right"[MeSH Terms] OR "ventricular function, left"[MeSH Terms] OR "Heart Failure"[MeSH Terms] OR "heart failure, diastolic"[MeSH Terms] OR "heart failure, systolic"[MeSH Terms] OR "Pulmonary Edema"[MeSH Terms] OR "Pneumothorax"[MeSH Terms] OR "Cardiac Tamponade"[MeSH Terms] OR "Pericardial Effusion"[MeSH Terms] OR "Pleural Effusion"[MeSH Terms] OR "Embolism and Thrombosis"[MeSH Terms] OR "Hemodynamics"[MeSH Terms] OR "Hemodynamic Monitoring"[MeSH Terms] OR "Hypotension"[MeSH Terms] OR "Intracranial Hypotension"[MeSH Terms] OR "Respiratory Insufficiency"[MeSH Terms] OR "Hypoxia"[MeSH Terms] OR "Oliguria"[MeSH Terms] OR "Acute Kidney Injury"[MeSH Terms] OR "Renal Insufficiency"[MeSH Terms] OR "Hyperemia"[MeSH Terms] OR "Intracranial Pressure"[MeSH Terms] OR "Intracranial Hypertension"[MeSH Terms] OR "Brain Edema"[MeSH Terms]) - Saved search Sort by: Most Recent | [2,217,849](https://pubmed.ncbi.nlm.nih.gov/searches/6860235/?mode=full&sort=date&size=100&long_term_hash=longquerya04a1640b2ea02f3ed66&ac=no) |
|  |  | #7 | ("Focused Assessment Sonography Trauma"[Title/Abstract:~2] OR "Focus-assessed transthoracic echocardiography"[Title/Abstract:~2] OR "Rapid Ultrasound Shock Hypotension"[Title/Abstract:~2] OR "Rapid Obstetric Screening Echocardiography"[Title/Abstract:~2] OR "Venous Excess Ultrasound"[Title/Abstract:~2] OR "Cardiac Arrest Sonographic Assessment"[Title/Abstract:~2] OR "Focused Echocardiography Emergency Life support"[Title/Abstract:~2] OR "Bedside Lung Ultrasound Emergency"[Title/Abstract:~2] OR "Rapid Assessment Dyspnea Ultrasound"[Title/Abstract:~2] OR "Focused Assessment Sonography Obstetrics"[Title/Abstract:~2]) OR ("Focused Assessment with Sonography for Trauma"[MeSH]) - Saved search Sort by: Most Recent | [853](https://pubmed.ncbi.nlm.nih.gov/searches/6860180/?mode=full&sort=date&size=100&ac=no) |
|  |  | #6 | ((((((((((((((((((((("optic nerve sheath diameter"[Title/Abstract:~3]) OR (transcranial*[Title/Abstract])) OR (Airway[Title/Abstract])) OR (larynx[Title/Abstract])) OR (laryng*[Title/Abstract])) OR (cricoid[Title/Abstract])) OR (cricothyroid[Title/Abstract])) OR ("tracheal rings"[Title/Abstract:~2])) OR (Cardiac[Title/Abstract])) OR (ventricular[Title/Abstract])) OR (valv*[Title/Abstract])) OR (gastric[Title/Abstract])) OR (antrum*[Title/Abstract])) OR (antral[Title/Abstract])) OR ("vena cava"[Title/Abstract:~3])) OR (IVC[Title/Abstract])) OR (vein[Title/Abstract])) OR (venous*[Title/Abstract])) OR (Arter*[Title/Abstract])) OR (kidney[Title/Abstract])) OR (renal*[Title/Abstract])) OR ("Ultrasonography, Doppler, Transcranial"[MeSH]) - Saved search Sort by: Most Recent | [3,617,603](https://pubmed.ncbi.nlm.nih.gov/searches/6860170/?mode=full&sort=date&size=100&ac=no) |
|  |  | #5 | ((POCUS[Title/Abstract]) OR (Point-of-care[Title/Abstract]) OR (bedside[Title/Abstract]) OR ("Point of care"[Title/Abstract]) OR (focused[Title/Abstract])) OR ("Point-of-Care Systems"[MeSH]) - Saved search Sort by: Most Recent | [456,766](https://pubmed.ncbi.nlm.nih.gov/searches/6852745/?mode=full&sort=date&size=100&ac=no) |
|  |  | #4 | #1 AND #2 AND #3 | [3,249](https://pubmed.ncbi.nlm.nih.gov/?term=%231+AND+%232+AND+%233&sort=relevance&size=100) |
|  |  | #3 | ((Anesthe*[Title/Abstract]) OR (critical care[Title/Abstract]) OR (Intensive care[Title/Abstract]) OR (Critically ill[Title/Abstract]) OR (Critical illness*[Title/Abstract]) OR (Anaesthe*[Title/Abstract])) OR ((((("Anesthetics"[MeSH]) OR "Anesthesia"[MeSH]) OR "Critical Care Outcomes"[MeSH]) OR "Critical Illness"[MeSH]) OR "Critical Care"[MeSH]) - Saved search Sort by: Most Recent | [782,160](https://pubmed.ncbi.nlm.nih.gov/searches/6852756/?mode=full&sort=date&size=100&ac=no) |
|  |  | #2 | (((((("Ultrasonography"[MeSH] OR "Ultrasonography, Doppler"[MeSH]) OR "Echocardiography"[MeSH]) OR "Echocardiography, Doppler"[MeSH]) OR "Echocardiography, Doppler, Color"[MeSH]) OR "Echocardiography, Transesophageal"[MeSH]) OR ((((((echocardiogr*[Title/Abstract]) OR (sonograph*[Title/Abstract])) OR (ultrasound[Title/Abstract])) OR (ultrasonogra*[Title/Abstract])) OR (echograph*[Title/Abstract])) OR (Doppler[Title/Abstract]))) - Saved search Sort by: Most Recent | [805,120](https://pubmed.ncbi.nlm.nih.gov/searches/6860152/?mode=full&sort=date&size=100&ac=no) |
|  |  | #1 | ((((((((((((((((((obstetric*[Title/Abstract]) OR (pregnan*[Title/Abstract])) OR (maternal[Title/Abstract])) OR (pre-eclamp*[Title/Abstract])) OR (preeclamp*[Title/Abstract])) OR (eclamp*[Title/Abstract])) OR ("gestational hypertension"[Title/Abstract])) OR ("hypertension in pregnancy"[Title/Abstract])) OR (gestational hypertension[Title/Abstract])) OR (intrapartum[Title/Abstract])) OR (postpartum[Title/Abstract])) OR (puerper*[Title/Abstract])) OR (cesarean[Title/Abstract])) OR (caesarean[Title/Abstract])) OR ("obstetric* shock"[Title/Abstract:~4])) OR (postpartum hemorrhage[Title/Abstract])) OR (postpartum haemorrhage[Title/Abstract])) OR ((((((("Postpartum Period"[MeSH]) OR "Perinatal Care"[MeSH]) OR "Pre-Eclampsia"[MeSH]) OR "Hypertension, Pregnancy-Induced"[MeSH]) OR "Pregnancy Complications"[MeSH]) OR "Obstetric Labor Complications"[MeSH]) OR "Pregnancy Complications, Cardiovascular"[MeSH])) - Saved search Sort by: Most Recent | [1,096,790](https://pubmed.ncbi.nlm.nih.gov/searches/6859190/?mode=full&sort=date&size=100&ac=no) |
