# Supplementary material for: Diagnostic point-of-care ultrasound in obstetric anesthesia and critical care: a scoping review protocol
Source: Syst Rev. 2024 Oct 24;13:268. doi: 10.1186/s13643-024-02673-3 (PMC11515486; doi:10.1186/s13643-024-02673-3)
Supplement: Supplementary file 2 — Supplementary Material 2: Supplementary file 2. A pilot comprehensive search in PubMed﻿ [file 13643_2024_2673_MOESM2_ESM.docx]

Supplementary file 2: Search Protocol, Strategy, and Terms

Search Protocol (PubMed, EMBASE, Web of Science):

1. Map each term to major subheading, then search each term separately using the specified parameters and combine using Boolean operator OR, repeating to form population, context and concepts 1-5 as described in “search parameters” (see below).
2. Combine “population OB” AND concept 1 (ultrasound) AND context anesthesia/critical care.
3. Combine concepts 2-5 that imply point-of-care uses using Boolean OR:

concept POC OR concept application OR concept protocol OR concept assessment.

1. Complete the search by combining search results from steps 2 AND 3
2. Limit/filter the final results to publication dates (Jan 1, 2000 – current), female, adult, English.

| **Initial Search Strategy Example (PubMed)** | |
| --- | --- |
| Search | **Query** |
| 1 | Obstetric* OR (Pregnan* OR (Postpartum OR pre-eclamp* OR preeclamp* OR gestational hypertension OR postpartum hemorrhage OR hypertension in pregnancy OR Eclamp* OR intrapartum OR puerperium OR Pregnancy complications[MeSH Terms] OR Pregnancy[MeSH Terms] OR Postpartum period[MeSH Terms] OR Postnatal care[MeSH Terms] OR Pre-eclampsia[MeSH Terms] OR Hypertension, Pregnancy Induced[MeSH Terms] |
| 2 | AND Echocardiogr* OR Sonograph* OR Ultrasound OR Ultrasonogra* OR Doppler OR Ultrasonography[MeSH Major Topic] OR Ultrasonography, Doppler[MeSH Major Topic] |
| 3 | Anesthe* OR critical care OR Intensive care OR Critical ill* OR Critically ill OR Anaesthe* OR Anesthesia[MeSH Terms] OR Anesthetics[MeSH Terms] OR Critical care[MeSH Terms] OR Critical illness[MeSH Terms] OR Critical care outcomes[MeSH Terms] |
| 4 | 1 AND 2 AND 3 |
| 5 | POCUS OR POC* OR Point-of-care OR Point of Care OR bedside OR Point of care systems [MeSH Terms] |
| 6 | optic nerve sheath diameter OR transcranial Doppler OR Airway ultraso* OR Airway sonogr* OR larynx ultraso* OR laryng* ultraso* OR larynx sonogr* OR laryng* sonogr* OR Cardiac ultraso* OR Cardiac sonogr* OR ventricular OR lung ultraso* OR lung sonogr* OR gastric ultraso* OR gastric sonogr* OR antrum* ultraso* OR antrum sonogr* OR TTE OR transthoracic echocar* OR transthoracic sonogr* OR transthoracic ultrasound* OR TEE OR transesophageal echocar* OR transesophageal ultraso* OR renal ultraso* OR renal sonogr* OR renal Doppler OR venous ultraso* OR venous sonogr* OR venous Doppler OR vein ultraso* OR vein sonogr* OR vein Doppler OR IVC ultraso* OR IVC sonogr* OR Inferior vena cava ultraso* OR Inferior vena cava sonogr* OR Ultrasonography, Doppler Transcranial[MeSH Terms] OR Echocardiography, transthoracic[MeSH Terms] OR Echocardiography, Doppler, Pulsed [MeSH Terms] OR Echocardiography, Doppler, Color[MeSH Terms] OR Echocardiography, transesophageal[MeSH Terms] OR Stroke Volume[MeSH Terms] |
| 7 | Focused Assessment Sonography Trauma **OR**  Focus-assessed transthoracic echocardiography **OR**  Rapid Ultrasound Shock Hypotension **OR**  Rapid Obstetric Screening Echocardiography **OR** “Venous Excess Ultrasound” OR Cardiac Arrest Sonographic Assessment **OR**  Focused Echocardiography Emergency Life support **OR**  Bedside Lung Ultrasound Emergency **OR**  Rapid Assessment Dyspnea Ultrasound **OR**  Focused Assessment Sonography Obstetrics **OR**  FASO **OR**  BLUE **OR**  FATE OR eFATE **OR** ROSE **OR** FEEL **OR** RADiUS **OR** VExUS **OR**  CASA **OR** I-AIM **OR** FAST OR eFAST **OR** Focused Assessment with Sonography for Trauma[MeSH Terms] |
| 8 | Stroke volume OR cardiac output OR ventric* function OR Contractil* OR Heart/cardiac/ventric* failure OR pulmonary edema OR pneumothorax OR tamponade OR effusion OR Emboli* OR Hemodynamic OR Hypotensi* OR Respiratory failure OR Hypoxi* OR Oliguri* OR renal failure OR venous congestion OR Intracranial hypertension OR Cerebral edema OR Cardiac Output[MeSH Terms] OR Stroke Volume([MeSH Terms] OR Ventricular Function[MeSH Terms] OR Ventricular Function, Right[MeSH Terms] OR Ventricular Function, Left[MeSH Terms] OR Heart Failure[MeSH Terms] OR Heart Failure, Systolic[MeSH Terms] OR Heart Failure, Diastolic[MeSH Terms] OR Pulmonary Edema[MeSH Terms] OR Pneumothorax[MeSH Terms] OR Cardiac Tamponade[MeSH Terms] OR Pericardial Effusion[MeSH Terms] OR Pleural Effusion[MeSH Terms] OR Embolism and Thrombosis[MeSH Terms] OR Hemodynamics[MeSH Terms] OR Hemodynamic Monitoring[MeSH Terms] OR Hypotension[MeSH Terms] OR Intracranial Hypotension[MeSH Terms] OR Respiratory Insufficiency[MeSH Terms] OR Hypoxia[MeSH Terms] OR Oliguria[MeSH Terms] OR Acute Kidney Injury[MeSH Terms] OR Renal Insufficiency[MeSH Terms] OR Hyperemia[MeSH Terms] OR Intracranial Pressure[MeSH Terms] OR Intracranial Hypertension[MeSH Terms] OR Brain Edema[MeSH Terms] |
| 9 | 5 OR 6 OR 7 OR 8 |
| 10 | 4 AND 9 |
| 11 | LIMIT to 2000-2023, adult, English, female |

| **SEARCH PARAMETERS** | **Population: obstetric** | **Concept 1: Ultrasound** | **Context:  anesthesia/critical care** | Terms that imply applications, protocols, and assessments in point-of-care ultrasound | | | |
| --- | --- | --- | --- | --- | --- | --- | --- |
|  |  |  |  | **Concept 2:  POC** | **Concept 3:  application** | **Concept 4: protocol** | **Concept 5:**  **assessment** |
| **keyword abstract title phrase (proximity)** | obstetric* | Echocardiogr* | anesthe*/anaesthe* | POCUS/POC* | optic nerve sheath diameter | Focused Assessment Sonography Trauma | stroke volume |
|  | pregnan* | Sonograph* | critical care | Point-of-care | transcranial Doppler | Focus-assessed transthoracic echocardiography | cardiac output |
|  | maternal | Ultrasound | Intensive care | “Point of care” | Airway/larynx ultraso*/ sonogr* | Rapid Ultrasound Shock Hypotension | ventric* function |
|  | pre-eclamp*/preeclamp* | Ultrasonogra* | critically ill | focused | cardiac ultraso*/ sonogr* | Rapid Obstetric Screening Echocardiography | Contractil* |
|  | eclamp* | Echograph* | critical illness* | bedside | lung ultraso*/ sonogr* | “Venous Excess Ultrasound” | Heart/cardiac/ventric* failure |
|  | gestational hypertension | Doppler |  |  | gastric/antrum ultraso*/ sonogr* | Cardiac Arrest Sonographic Assessment | pulmonary edema |
|  | “hypertension in pregnancy” |  |  |  | TTE | Focused Echocardiography Emergency Life support | pneumothorax |
|  | intrapartum |  |  |  | transthoracic echocar*/ sonogr* | Bedside Lung Ultrasound Emergency | tamponade |
|  | postpartum |  |  |  | transthoracic ultrasound* | Rapid Assessment Dyspnea Ultrasound | effusion |
|  | puerper* |  |  |  | TEE | Focused Assessment Sonography Obstetrics | Emboli* |
|  | cesarean/caesarean |  |  |  | Transesophageal echocar*/ultraso* | FASO | Hemodynamic |
|  | obstetric* shock |  |  |  | renal ultraso*/sonogr*/Doppler | BLUE | Hypotensi* |
|  | postpartum hemorrhage/haemorrhage |  |  |  | Venous ultraso*/ sonogr*/Doppler | FATE/eFATE | Respiratory failure |
|  |  |  |  |  | Vein ultraso*/sonogr*/Doppler | ROSE | Hypoxi* |
|  |  |  |  |  | IVC ultraso*/sonogr* | FEEL | Oliguri* |
|  |  |  |  |  | Inferior vena cava ultraso*/ sonogr* | RADiUS | renal failure |
|  |  |  |  |  |  | VExUS | venous congestion |
|  |  |  |  |  |  | CASA | Intracranial hypertension/pressure |
|  |  |  |  |  |  | I-AIM | Cerebral edema |
|  |  |  |  |  |  | FAST |  |
|  |  |  |  |  |  | eFAST |  |
| **MeSH**  **topic heading**  **subheading** | Pregnancy (MeSH) | Ultrasonography, Doppler (MeSH) | Anesthetics (MeSH) | Point of care systems (exploded to include Point of care testing) (MeSH) | Ultrasonography, Doppler Transcranial(MeSH) | Focused Assessment with Sonography for Trauma(MeSH) | Cardiac Output(MeSH) |
|  | Postpartum period (MeSH) | Ultrasonography (MeSH) | Anesthesia (MeSH) |  | Echocardiography, transthoracic(MeSH) |  | Stroke Volume(MeSH) |
|  | Perinatal care (MeSH) |  | Critical care outcomes (MeSH) |  | Echocardiography, Doppler, Pulsed(MeSH) |  | Ventricular Function(MeSH) |
|  | Pre-eclampsia (MeSH) |  | Critical illness (MeSH) |  | Echocardiography, Doppler, Color(MeSH) |  | Ventricular Function, Right(MeSH) |
|  | Hypertension, Pregnancy Induced (MeSH |  | Critical care (MeSH) |  | Echocardiography, Transesophageal(MeSH) |  | Ventricular Function, Left(MeSH) |
|  | Pregnancy complications (MeSH) |  |  |  | Stroke Volume(MeSH) |  | Heart Failure(MeSH) |
|  | Obstetric Labor Complications(MeSH) |  |  |  |  |  | Heart Failure, Systolic(MeSH) |
|  |  |  |  |  |  |  | Heart Failure, Diastolic(MeSH) |
|  | Pregnancy Complications, Cardiovascular(MeSH) |  |  |  |  |  | Pulmonary Edema(MeSH) |
|  |  |  |  |  |  |  | Pneumothorax (MeSH) |
|  |  |  |  |  |  |  | Cardiac Tamponade (MeSH) |
|  |  |  |  |  |  |  | Pericardial Effusion (MeSH) |
|  |  |  |  |  |  |  | Pleural Effusion (MeSH) |
|  |  |  |  |  |  |  | Embolism and Thrombosis (MeSH) |
|  |  |  |  |  |  |  | Hemodynamics (MeSH) |
|  |  |  |  |  |  |  | Hemodynamic Monitoring (MeSH) |
|  |  |  |  |  |  |  | Hypotension  (MeSH) |
|  |  |  |  |  |  |  | Intracranial Hypotension  (MeSH) |
|  |  |  |  |  |  |  | Respiratory Insufficiency (MeSH) |
|  |  |  |  |  |  |  | Hypoxia (MeSH) |
|  |  |  |  |  |  |  | Oliguria (MeSH) |
|  |  |  |  |  |  |  | Acute Kidney Injury (MeSH) |
|  |  |  |  |  |  |  | Renal Insufficiency (MeSH) |
|  |  |  |  |  |  |  | Hyperemia (MeSH) |
|  |  |  |  |  |  |  | Intracranial Pressure (MeSH) |
|  |  |  |  |  |  |  | Intracranial Hypertension (MeSH) |
|  |  |  |  |  |  |  | Brain Edema (MeSH) |

* MeSH major headings are specific to PubMED. We will use Emtree major headings when searching EMBASE. Web of Science Core Collection does not use major headings and we will use MeSH terms as phrases in addition to individual terms.
