# Supplementary figures and images for: Diagnostic point-of-care ultrasound in obstetric anesthesia and critical care: a scoping review protocol
Source: Syst Rev. 2024 Oct 24;13:268. doi: 10.1186/s13643-024-02673-3 (PMC11515486; doi:10.1186/s13643-024-02673-3)

Supplementary file 3: PRISMA Flow-chart


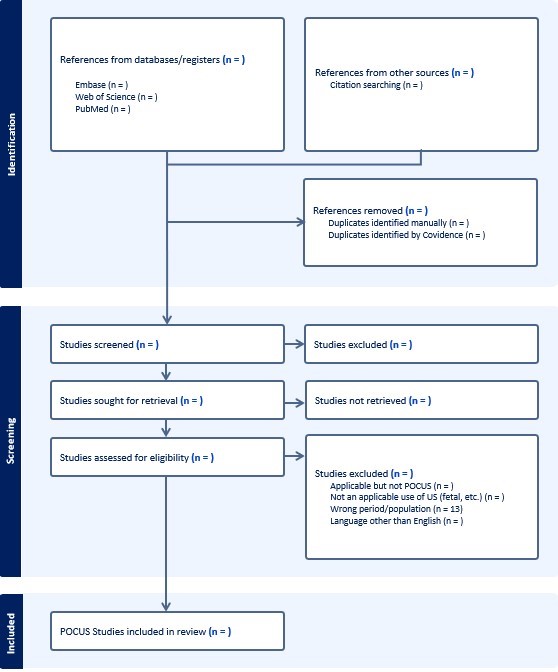

Supplement: Supplementary file 3 — Supplementary Material 3: Supplementary file 3. PRISMA Flow-chart [file 13643_2024_2673_MOESM3_ESM.docx]
