# Supplementary material for: Diagnostic point-of-care ultrasound in obstetric anesthesia and critical care: a scoping review protocol
Source: Syst Rev. 2024 Oct 24;13:268. doi: 10.1186/s13643-024-02673-3 (PMC11515486; doi:10.1186/s13643-024-02673-3)
Supplement: Supplementary file 4 — Supplementary Material 4: Supplementary file 4. POCUS in obstetric anesthesia data extraction instrument and codebook with guidance [file 13643_2024_2673_MOESM4_ESM.docx]

Supplementary file 4: POCUS in obstetric anesthesia data extraction instrument and codebook with guidance.

| **variable** | **type** | **units** | **values** | **labels** | **note** |
| --- | --- | --- | --- | --- | --- |
| **id** | num (integer) |  |  | Article | Assigned identifier for each included article |
| **PUBLICATION CHARACTERISTICS** | | | | | |
| **auth** | string |  |  | Primary author | Last name of the primary author |
| **title** | string |  |  |  | Article title |
| **year** | num | time |  | Year | Year of publication |
| **country** | string |  |  | Country | Country of publication |
| **journal** | string |  |  | Journal | Journal of publication |
| **impact** | continuous |  |  | Impact factor | Journal impact factor |
| **citations** | num (integer) | count |  | Citations | Number of article citations at the time of the literature search. |
| **Alt** | continuous |  |  | Altmetric | Altmetric score for the article at the time of the literature search. |
| **terminology** | categorical |  | 1  2 | POCUS  other | What terminology does the publication use? |
| **METHODOLOGY** | | | | | |
| **studysize** | num (integer) | count |  | sample size | Total number of participants/patients in the study. |
| **methodology** | categorical |  | 1  2  3  4 5  6  7 8  9  10  11  12 | interventional  observational prospective  observational retrospective  cross-sectional  review -  systematic/meta  review - narrative feasibility reliab/reproducibility  case series case report  editorial other |  |
| **comparison** | categorical |  | 0  1 | no yes | Did the study include a parallel arm or another group for comparison? |
| **applicable** | binary |  | 1  2 | typical applicable | Does the study describe a typical application of POCUS in OB anesthesia/critical care or is it applicable to POCUS? For example, a study of association between MAPSE and diastolic dysfunction in preeclampsia that was done in an echo lab is not typical POCUS but the findings would be applicable to POC use. |
| **future** | narrative |  |  | Future directions | Narrative description of future directions for research/implementation |
| **notes** | narrative |  |  |  | Any relevant notes about the study. |
| **CLINICAL ASPECTS** | | | | | |
| **reason** | string |  | 1  2  3  4  5  6  7  8  9  10 | Intervention  Measure outcome  Measure exposure  Reduce diagnostic uncertainty  Expedite decision-making  Monitor  Accuracy/validation  Describe technique(s)  Discuss application(s) | Describe the primary utility of POCUS in the article. Multiple selections possible. |
| **application** | categorical |  | 1  2  3  4  5  6  7  8  9  10  11 | cardiac lung gastric airway ONSD venous arterial  transcranial abdominal  renal  other | Sonographic focus of the article. Studies may include multiple end-organs as ultrasound targets. |
| **indication** | categorical |  | 1  2  3  4  5  6  7  8  9  10  11  12  13 | Hemodynamic instability/shock  Hypox/dyspnea  Chest pain  Oliguria  Decreased LOC  Headache  Seizure  Aspiration risk/ emergency GA  Potential diff airway  FONA  Esophageal intubation  Polytrauma  Other | Which clinical condition was the reason US was used? Multiple selection enabled. |
| **ind** | narrative |  |  | Other indications | Describe indications other than the primary or inclusion criteria. |
| **assessment** | categorical |  | 1  2  3  4  5  6  7  8  9  10  11  12  13  14  15  16  17 | Intravasc volume Cardiac output Ventricular function  Pericardial effusion  Embolism (DVT, PE, AFE or VAE)  Lung water  Pleural effusion  Pneumothorax  Lung consolidation  Peritoneal fluid/blood  Gastric contents qualitative  Gastric contents quantitative  Cerebral edema  Increased ICP  Identify difficult airway  Identify structures for FONA  Other | What was the US assessment used or described? Studies/cases may utilize more than one assessment. For example, in hemodynamic instability (indication), options 1-8 and 10 may be relevant. |
| **assessment** | narrative |  |  |  | If above categories incompletely capture the assessment, describe what was proposed/described. |
| **protocol** | categorical |  | 1  2  3  4  5  6  7  8  9  10  11 | FAST/eFAST  FoCUS  FATE  ROSE  FEEL  FASO  RADiUS  VExUS  CASA  BLUE  n/a | Which of the established protocols were featured in the article? |
| **utility** | categorical |  | 1    2  3  4  5  6  7 | change clinical outcome  change management  change time to management or outcome  contribute to understanding of pathophysiology  optimize resource utilization  rule out a differential diagnosis  other  N/A | For articles with clinical indications, what was the primary effect of the use of POCUS? How did POCUS use make a difference?  Studies where POCUS was used to re-classify risk align with option 2. An article that shows POCUS can bypass CT scan for suspected PE would be an example of options 3 and 5, and potentially 2. |
| **TECHNICAL ASPECTS** | | | | | |
| **usmode** | categorical |  | 1  2  3  4 5  6  7  8 | B mode (2D)  M mode  Color Doppler  Spectral Doppler  Power Doppler  Speckle tracking  3D/4D  Not specified | Which ultrasound mode was used in the article? Multiple selection. |
| **technology** | categorical |  | 1  2  3  4 | Stand-alone  portable  hand-held (ultraportable)  not specified | Manufacturer’s description may be necessary if not stated in the article. |
| **probe** | categorical |  | 1  2  3  4  5 | linear  curvilinear  phased array  TEE  Not specified | Multiple selection. |
